# Resonant Metasurfaces with Van Der Waals Hyperbolic Nanoantennas and Extreme Light Confinement

**DOI:** 10.3390/nano14181539

**Published:** 2024-09-23

**Authors:** Viktoriia E. Babicheva

**Affiliations:** Department of Electrical and Computer Engineering, MSC01 11001, University of New Mexico, Albuquerque, NM 87131, USA; vbb@unm.edu

**Keywords:** hexagonal boron nitride, directional scattering, hyperbolic dispersion, Reststrahlen bands, optical antenna, multipolar resonances

## Abstract

This work reports on a metasurface based on optical nanoantennas made of van der Waals material hexagonal boron nitride. The optical nanoantenna made of hyperbolic material was shown to support strong localized resonant modes stemming from the propagating high-k waves in the hyperbolic material. An analytical approach was used to determine the mode profile and type of cuboid nanoantenna resonances. An electric quadrupolar mode was demonstrated to be associated with a resonant magnetic response of the nanoantenna, which resembles the induction of resonant magnetic modes in high-refractive-index nanoantennas. The analytical model accurately predicts the modes of cuboid nanoantennas due to the strong boundary reflections of the high-k waves, a capability that does not extend to plasmonic or high-refractive-index nanoantennas, where the imperfect reflection and leakage of the mode from the cavity complicate the analysis. In the reported metasurface, excitations of the multipolar resonant modes are accompanied by directional scattering and a decrease in the metasurface reflectance to zero, which is manifested as the resonant Kerker effect. Van der Waals nanoantennas are envisioned to support localized resonances and can become an important functional element of metasurfaces and transdimensional photonic components. By designing efficient subwavelength scatterers with high-quality-factor resonances, this work demonstrates that this type of nanoantenna made of naturally occurring hyperbolic material is a viable substitute for plasmonic and all-dielectric nanoantennas in developing ultra-compact photonic components.

## 1. Introduction

Optical antennas have great potential for nanophotonic applications that rely on effective light localization and management, the strong interaction between light and matter, improved energy concentration, optimized light collection, and related phenomena [1,2]. Plasmonic structures have been investigated for a broad spectrum of functionalities, and plasmonic antennas have brought promising ways to localize light on a subwavelength scale and move toward miniaturization [3,4,5], including practical applications of sensors and near-field optical microscopy [6,7,8]. Subwavelength plasmonic nanoantennas of basic shapes, such as spheres or disks, exhibit primarily electric dipolar and quadrupolar resonant excitations. Generating a magnetic resonance in the metasurface involves designing more intricate structures and engineered arrangements, including core-shell nanoantennas, split-ring nanoresonators, or antennas featuring gaps and interlays of metal and dielectrics. To address these limitations, nanoantennas composed of metal–dielectric nanostructures made up of multiple and repetitive layers have been suggested, and the resonant states of both electric and magnetic multipoles have been investigated [9,10,11]. However, the consistent and large-scale realization of such nanoantennas faces enormous challenges, and alternative approaches have been sought. In turn, high-refractive-index nanoantennas, particularly those made of silicon, have generated significant interest and have arisen as a viable substitute for plasmonic nanostructures [12,13,14,15]. Nanoantennas of simple shapes support both electric and magnetic resonant excitations, and resonant modes can overlap at specific wavelengths or in a broader range when excited in disk nanoantennas, resulting in directional scattering [16].

Both plasmonic and all-dielectric nanostructures present functional limitations and barriers to practical applications in photonics and optical devices. Plasmonic nanostructures are made of metals and are prone to nonradiative loss and heat dissipation. In high-refractive-index nanostructures, such as silicon, III-V compounds, and germanium, nonradiative losses are negligible due to the low dissipation in the constituent material for the photon energies below the bandgap. However, the radiative losses are high, dominant at the subwavelength scale, and prevent photonic systems from scaling down. Radiation-based loss is greater in nanostructures made of materials with moderate values of refractive indices, such as metal oxides [17].

Optical nanoantennas composed of naturally occurring material with hyperbolic dispersion have attracted much attention [18,19]. In particular, there is an interest in hexagonal boron nitride (hBN), which has been shown to be a hyperbolic medium in particular frequency bands because of phonon-polariton excitations. The properties of the hyperbolic medium stem from opposing signs in the real part of diagonal permittivity components, leading to the hyperbolic dispersion of propagating waves with respect to frequency. Because of the hyperbolic dispersion, the hyperbolic medium supports waves with a large wavevector (high-k waves), which enables a number of intriguing phenomena, such as enhanced and spectrally broad spontaneous emission, anomalous heat transfer, etc. [20,21,22,23,24,25,26].

The strong localization of wave propagating in a hyperbolic medium has been explored in waveguides [27,28,29,30] and tapers [31]. Artificially engineered hyperbolic media (hyperbolic metamaterials or HMMs) have been investigated, and two primary methods for HMM fabrication are gaining popularity: metal nanorod arrays and metal–dielectric multilayer configurations. HMM-based cavities have been demonstrated to resonate with various field configurations and states [32,33,34,35,36,37], which can result in an improvement in radiation efficiency, spasing, etc. [38,39,40,41,42,43,44]. Due to the metallic constituents in these nanostructures, optical losses in these constituents are high, the response is not spectrally localized, and, as a consequence, the performance of such effective-medium resonators is moderate. Another interesting direction involves epitaxially grown III-V semiconductor materials with different layer doping concentrations in a multilayer structure, which can effectively result in hyperbolic dispersion [45,46]. This approach has several advantages, such as the possibility to produce thin, smooth layers and tune their permittivity in a relatively wide range. However, such multilayer structures are essentially composites, and their realization is more difficult in comparison to processing bulk material with natural hyperbolic dispersion.

Recently, polar dielectrics, such as hBN [47,48,49,50,51,52,53,54], molybdenum trioxide (α-MoO_3_) [55,56,57], silicon carbide [58,59], and gallium nitride [60], have been demonstrated to possess low-loss phonon-polariton resonant modes in the mid-infrared range. These resonances result in spectral bands, also known as Reststrahlen bands, in which at least one component of the permittivity tensor is negative and enable optical phenomena analogous to those in plasmonic nanostructures, but with significantly lower nonradiative dissipation [61]. In polar dielectric materials, polarization results from oscillated ions as opposed to electrons in metals. The lifetime of these ion oscillations (phonon polaritons) can be greater than the lifetime of plasmonic oscillations (plasmon polaritons) because of the substantially longer timescale of the scattering. Recently emerged ternary van der Waals crystals have been demonstrated to possess giant in-plane optical anisotropy in the spectral range from visible to mid-infrared [62].

Layered materials and their heterostructures hold great promise for transdimensional photonics [63]. Graphene-based hyperbolic metamaterials are paving the way for transdimensional photonics, enabling the design of cutting-edge nanophotonic devices with adaptable features. In the mid-infrared spectrum, a polarization-sensitive tunable hyperbolic microcavity has been realized through precise control of the thickness of the dielectric layers, breaking the regularity of the graphene-based metamaterial stack and introducing dynamic control across different spatial dimensions [64]. This approach highlights the potential of transdimensional photonics for creating reconfigurable optical systems made of hyperbolic cavities. Furthermore, a hyperbolic metamaterial made of thin layers of indium tin oxide and silicon dioxide has been theoretically demonstrated to feature a tunable canalization wavelength in the near-infrared range [65]. By leveraging the principles of transdimensional photonics, this system offers precise control over light propagation, enabling enhanced manipulation of light through the structure.

Thin layers of naturally occurring hyperbolic material hBN can serve as key elements in next-generation compact photonic technologies and optical systems [66,67,68,69,70,71,72,73,74,75]. Of all of the polar dielectrics investigated so far, hBN has the most intriguing properties, and hBN nanoparticles have been suggested as subwavelength nanoresonators [76,77,78,79,80,81]. Hexagonal boron nitride layers are bound by weak van der Waals forces, and because of the layered structure of hBN, in-plane and out-of-plane permittivity-component resonances are excited at two distinct spectral bands. These two distinct Reststrahlen bands range in wavelength from approximately 6.2–7.4 µm to 12.2–13.2 µm. As a result, bulk hBN has a natural hyperbolic dispersion with low nonradiative losses because of the large lifetime of phonon polaritons.

The analysis of resonant metasurfaces with van der Waals hyperbolic nanoantennas is driven by the need to engineer precise light-matter interactions at the nanoscale, exploiting the unique optical properties of two-dimensional and layered materials. Hyperbolic dispersion in van der Waals materials enables extreme confinement and directional propagation of light, enhancing optical responses in subwavelength structures. The resonant behavior of nanoantennas in these metasurfaces is carefully tuned by leveraging anisotropic permittivity, enabling new avenues for controlling near-field and far-field optical waves. These advances hold potential for applications in nanoscale photonic devices, where controlling light beyond the diffraction limit is critical.

In this work, van der Waals hBN is shown to serve as a potential replacement for plasmonic and all-dielectric materials in the development of optical metasurfaces and nanoantennas, constituting photonic metasurfaces as building blocks. The resonance results from the strong reflection of these waves from the nanoantenna edges. Due to the high-k internal waves and their strong reflection and bouncing from the edges of the cuboid nanoantennas, the modes are accurately predicted by the analytical model. This approach, however, is not applicable to plasmonic or high-refractive-index nanoantennas, where the weakened boundary reflection leads to more complex interactions that the analytical model cannot adequately address. The work reports on the scattering properties of hBN nanoantennas, and they are demonstrated to exhibit electric and magnetic resonant states. Overlaps in the wavelength of these excitations can result in unidirectional scattering (the Kerker effect), and a rectangular array of the nanoantennas in a lattice can result in collective resonances. For the cuboid nanoantenna with a hyperbolic response, the simulation results demonstrate a number of multipolar resonant modes, a decrease in reflectance of the metasurface, and highly directional resonant scattering from nanoantenna pairs. The effects demonstrated in this work lay the groundwork for advancing the design of functional metasurfaces and ultrathin optical components based on van der Waals heterostructures.

## 2. Cuboid Model and Resonances

Full-wave numerical simulations were conducted using a frequency-domain solver in the commercial software CST Studio Suite (Version 2024). The solver uses a finite-element-method-based approach to solve Maxwell’s equations, and it shares similarities with conventional solvers by employing tetrahedral meshing, which is particularly effective for complex geometries. It distinguishes itself through adaptive mesh refinement and efficient algorithms optimized for high-frequency range. Due to the adaptive mesh refinement and tetrahedral meshing employed by the solver, specifying a fixed grid size is not feasible; however, thorough testing confirms that the results are robust and do not change with varying mesh resolutions, ensuring reliable convergence.

**Figure 1:**
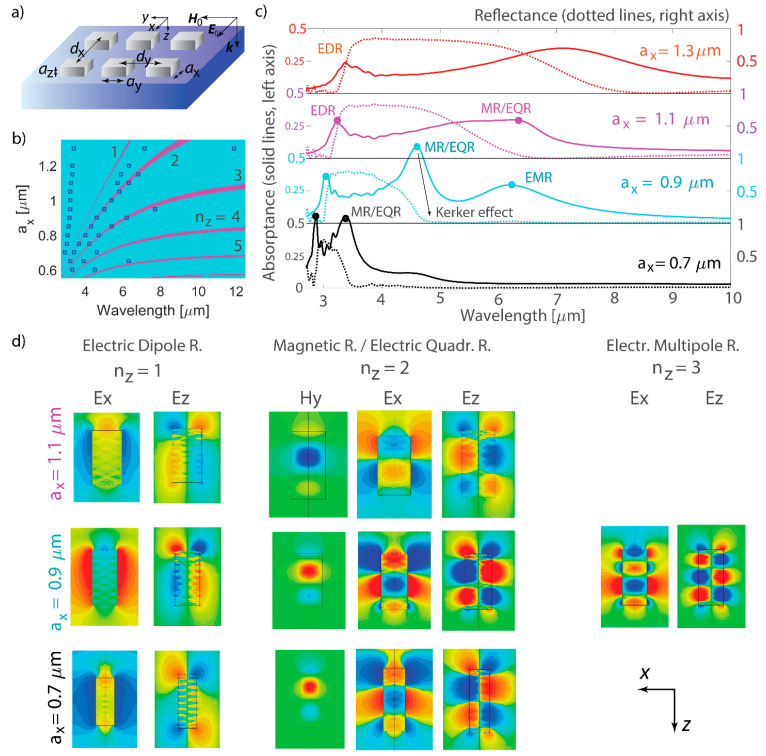
Multipolar resonant modes in hyperbolic-medium nanoantennas. (**a**) Schematic view of the metasurface: the cuboid nanoantennas have side lengths *a*_x_ × *a*_y_ × *a*_z_, organized in the array with the pitches *d*_x_ and *d*_y_, and the incident plane wave has *x*-polarization. (**b**) *n*_z_ is dependent on *a*_x_ according to Equation (2). The dimensions of the nanoantenna are *a*_y_ = 0.5 µm and *a*_z_ = 2 µm with excitation being *n*_y_ = 0 and *n*_x_ = 1. The blue square marks indicate the resonant excitations observed in the full-wave simulation. (**c**) Full-wave simulations of the absorptance and reflectance for the varying *a*_x_. Dots indicate absorptance maxima where the electromagnetic field maps are taken for electric dipolar (denoted ‘EDR’), electric quadrupolar (denoted ‘EQR’) and magnetic (denoted ‘MR’), and electric multipolar resonances (denoted ‘EMR’). The metasurface pitches are *d*_x_ = 2.5 µm and *d*_y_ = 2.5 µm, and the lengths of the nanoantenna are *a*_y_ = 0.5 µm and *a*_z_ = 2 µm. (**d**) Mapping of the electric *E*_x_ and *E*_z_ and magnetic *H*_y_ components corresponding to the resonant excitations. The magnetic component is plotted for *n*_z_ = 2, when the resonant magnetic mode is excited. The XZ coordinate plane is shown for nanoantenna cross-sections.

The optical properties of a metasurface consisting of a nanoantenna array with pitches *d*_x_ and *d*_y_ were investigated under illumination with an *x*-polarized plane wave (Figure 1a). Unless stated otherwise, a plane wave is normally incident on the array and propagates along the z-axis. For oblique incidence, we consider TM polarization, i.e., having field components (*E*_x_, *H*_y_, *E*_z_). The frequency sampling is dense enough to capture all spectral features accurately, with only linear interpolation between the discrete frequency points applied, avoiding any approximations, smoothing, or higher-order interpolation techniques.

A nanoantenna in the unit cell was modeled with periodic boundary conditions in the *x*- and *y*-axes, mimicking an infinite array. The built-in Floquet–Bloch boundary conditions were employed to model periodic structures by enforcing phase continuity across boundaries, ensuring that the electromagnetic fields exhibit periodic variation in the spatial domain. This allowed for accurate simulation of wave propagation in periodic structures using a frequency-domain solver, including the case of oblique incidence. The effect of infinite uniform surrounding was achieved by using sufficiently thick artificial perfectly matched layers on the *z*-axis. Ports were placed along the *z*-axis with a spacing of 60 μm, where they acted as excitation and detection planes for propagating waves. The peaks in the absorptance spectra agree well with the resonance excitations of a single nanoantenna when the array period is smaller than the wavelength (*d*_x_, *d*_y_ < λ), and the lattice effect can be neglected. Nanoantennas were cuboids with dimensions *a*_x_, *a*_y_, and *a*_z_ and were embedded in free space (modeled as uniform material with refractive index *n*_e_ = 1).

The nanoantenna material has hyperbolic properties and permittivity tensor terms Re(ε_y_,ε_x_) < 0 and Re(ε_z_) > 0 (type II hyperbolicity, considering ε_x_ = ε_y_). The response of nanoantennas corresponds to hBN at λ ≈ 7 µm with in-plane components ε_x_ = ε_y_ = −14.6 + 1.0i and out-of-plane component ε_z_ = 2.70 [82]. Resonances in nanoantennas are induced by the reflection of waves from their edges [83,84]. To demonstrate the broader concept of nanoresonator excitation and design a strategy for the cuboid nanoantenna, the effect of wavelengths with fixed permittivity value was analyzed. Subsequently, the properties of nanoantennas with a spectrally dependent hBN response were simulated.

In type II HMM, the wavevector (*k*_x_, *k*_y_, *k*_z_) of the propagating mode obeys the equation [48]

$$\frac{k_{x}^{2}+k_{y}^{2}}{\varepsilon_{z}}+\frac{k_{z}^{2}}{\varepsilon_{x}}=k_{0}^{2},$$

where *k*_0_ = 2π/λ_0_ and λ_0_ is the free-space wavelength of incident light. It is hypothesized and subsequently confirmed that in the cuboid nanoantenna with size lengths *a*_x_, *a*_y_, and *a*_z_, the resonant modes are excited when

(1) $$k_{x}a_{x}=\pi n_{x},\;k_{y}a_{y}=\pi n_{y},\;k_{z}a_{z}=\pi n_{z},$$

where *n*_x_, *n*_y_, and *n*_z_ are integers. Therefore, under resonant excitation, the relation between the nanoantenna dimensions and permittivity components is

(2) $$\frac{1}{\varepsilon_{z}}{\left(\frac{n_{x}}{2a_{x}}\right)}^{2}+\frac{1}{\varepsilon_{z}}{\left(\frac{n_{y}}{2a_{y}}\right)}^{2}+\frac{1}{\varepsilon_{x}}{\left(\frac{n_{z}}{2a_{z}}\right)}^{2}=\frac{1}{\lambda_{0}^{2}}.$$

In a broader context, Equation (1) should include phase changes at the nanoantenna boundaries ϕ_0_, for example $k_{x}a_{x}+2\varphi_{0}=\pi n_{x}$. However, here, the focus is on the most basic model and on the demonstration of its effectiveness. As a result of the opposite signs of the permittivity tensor components Re(ε_x_) and Re(ε_z_), the wavenumbers *k*_x_ and *k*_z_ can become extremely large (consistent with earlier discussed high-k waves in HMM). The most important point to emphasize here is that because of the excitation of such high-k waves, even with deeply subwavelength dimensions, the hyperbolic-medium nanoantenna can support a number of different resonances, including not only an electric dipolar, but also a magnetic dipolar, an electric quadrupolar, and higher multipolar modes.

Upon changing one cuboid dimension *a*_x_, the resonances were excited at different spectral positions. Model Equation (2) allows for calculations of resonance positions (Figure 1b). In addition, modeling of the reflectance and absorptance spectra was performed for the nanoantenna array, and resonance positions were identified (Figure 1c). A comparison of the results obtained from Equation (2) and full-wave modeling shows good agreement, and only resonances at shorter wavelengths were not predicted by the model. Excited resonant modes can be categorized based on *n*_z_ (Figure 1d): *n*_z_ = 1 indicates electric dipolar (EDR), *n*_z_ = 2 corresponds to electric quadrupolar (EQR), and excitations with *n*_z_ ≥ 3 are electric multipolar resonances (EMRs). The MR was excited more efficiently with *n*_z_ = 2, and the map of the H_y_ component in the resonant mode was presented in those cases only (see Figure 1d). The strongest magnetic resonances were identified with *n*_z_ = 2. Derivations of *n*_z_ with Equation (2) offer a broad framework for predicting the excitations of electric and magnetic resonant modes in a nanoantenna of specific size length. Only EDRs deviate from the equation predictions and typically emerge at shorter wavelengths. On the contrary, higher multipolar excitations emerge at longer wavelengths, which differs from plasmonic and high-refractive-index nanoantenna modes, where the higher multipolar response is confined to a narrower wavelength band due to the anomalous scaling law in metal–dielectric nanostructures [33].

The mechanism of magnetic resonance (MR) excitation is the same for high-refractive-index nanoantennas: repetitive bouncing of the wave from the nanoantenna edges induces circulation of the E-field inside the nanoantenna, and thus MR is induced. This mechanism is drastically different from the excitation of magnetic resonances in plasmonic nanoantennas. In the case of a nanoantenna with either negative permittivity and small losses or permittivity of any sign and high losses, the field does not penetrate inside the nanoantenna and is mainly localized outside the nanoantenna. To achieve a considerable magnetic response from the plasmonic nanoantenna, the introduction of a ‘gap’ or multilayer structure between metal parts or the design of split-ring resonators is required. High-refractive-index nanoantennas have been shown to overcome this limitation because of the field penetration inside the nanoantenna and the possibility of electric field circulation resulting in a magnetic response. Supporting high-k waves, the hyperbolic-medium nanoantenna provides a significant magnetic response that can be used along with the electric resonances to control the direction of light scattering and reflectance of the array.

This importance of reflection from nanoantenna boundaries has been well-established for dielectric materials, where high-quality-factor cavities with controlled quality factors and reduced radiative loss can be designed on a subwavelength scale. Nanoantennas made from materials with moderate refractive indices, such as titanium dioxide, sapphire, or cupric oxide, generally do not support particularly strong resonances. In contrast, strong resonances are typically achieved with high-refractive-index materials such as silicon, germanium, or III-V compounds. Materials with high-k waves, characterized by their large wavevectors and strong boundary reflections, represent the next step in advancing nanoantenna design by enabling even more precise mode confinement and enhanced resonant properties.

It is crucial to emphasize that the analytical model accurately predicts the modes of cuboid nanoantennas due to strong high-k wave reflections, resulting in high-quality-factor cavities with minimal radiative loss. This capability stems from the cuboid’s effective confinement of waves and low mode leakage. Such an analytical approach, however, does not extend to plasmonic or high-refractive-index nanoantennas, where the complex interactions and significant radiative losses complicate the analysis. This aspect has not been utilized in previous analyses because it is specific to cuboid nanoantennas made of material supporting high-k waves and does not apply to nanoantennas made of other materials. The analogy between a cuboid nanoantenna and a cavity highlights how the former achieves efficient mode confinement and low leakage, differentiating it from other nanoantennas where this model is not applicable.

## 3. Scattering and Interference

The conventional Kerker effect can be observed when the electric and magnetic dipolar polarizabilities are equal in phase and magnitude [85,86]. In this case, the light scattered backwards by each multipole interferes destructively, and the backward scattering is zero or significantly suppressed [86,87,88,89]. More complex scattering processes involve higher multipoles, such as an electric quadrupole, and the effect of backward scattering suppression is commonly referred to as the generalized Kerker effect [90,91]. Not only the shape of the nanoantenna but also its arrangement in the lattice can play an important role in resonance excitation and result in the lattice Kerker effect appearing as a zero reflectance of the nanoantenna array [92]. In the case shown in Figure 1c, the excitation of MR/EQR leads to a significant decrease in reflectance to nearly zero, caused by the destructive interference of backward-scattered light by EDR, EQR, and MR, and the generalized Kerker effect. For spherical nanoantennas with scalar polarizability, the arrangement of the array has been shown to facilitate coupling between EQR and MR [93], and resonances can either enhance or suppress each other. In the cuboid nanoantenna, MR and EQR couple to each other and coincide with EDR, fulfilling the generalized Kerker condition.

**Figure 2:**
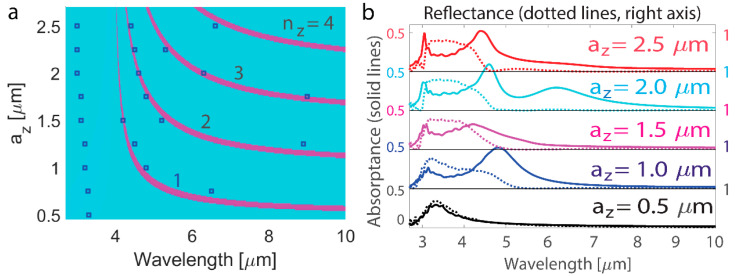
Effects of *a*_z_ and *n*_z_ on the resonance position in the nanoantenna array. (**a**) Comparison of *n*_z_ obtained with Equation (2) for varying *a*_z_ (color map) and with full-wave simulations (blue marks). The dimensions of the nanoantenna are *a*_x_ = 0.9 µm and *a*_y_ = 0.5 µm, and excitations with *n*_y_ = 0, and *n*_x_ = 1 are considered. (**b**) Numerical calculations of the absorptance and reflectance spectra of the nanoantenna array for varying *a*_z_. The metasurface arrangement is with *d*_x_ = 2.5 µm and *d*_y_ = 2.5 µm, and the nanoantennas have dimensions of *a*_x_ = 0.9 µm and *a*_y_ = 0.5 µm. The blue marks in panel a are defined as the maximum absorptance values obtained in numerical modeling.

Similarly to the variations of transverse cuboid dimensions, changes of resonance for the varying dimension along the light incidence *a*_z_ are analyzed. Apart from resonances with *n*_z_ = 1, 2, 3, …, which all agree well with the results of numerical modeling, an additional resonance at a shorter wavelength is excited (Figure 2a). The reflectance and absorptance spectra are similar to those of the previous case with *a*_x_ variations, and significant drops in reflectance are observed at the position where resonances overlap and are out-of-phase (Figure 2b). The effective medium model has predicted resonant modes in waveguides made of an effectively hyperbolic medium with a rectangular cross-section [27], wire nanoresonators [32], and artificial hyperbolic-medium spherical particles [84]. Fabry–Perot resonant modes have been shown for subwavelength dielectric bars [85].

**Figure 3:**
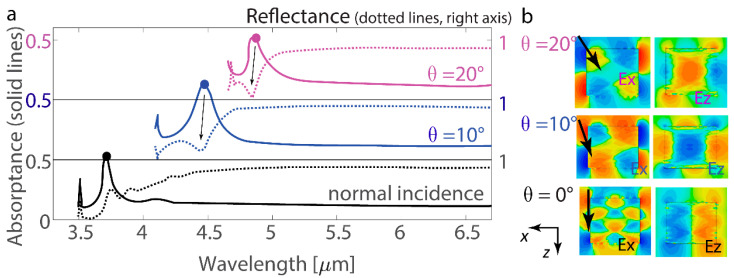
Oblique light incidence on the nanoantenna array. (**a**) Absorptance and reflectance spectra (solid and dotted lines, respectively) of the nanoantenna array at various angles of incident light with TM polarization. The dots demonstrate maxima where the electric field maps are plotted. The metasurface arrangement is with *d*_x_ = 3.5 µm and *d*_y_ = 2.5 µm, and the nanoantennas have dimensions of *a*_x_ = 2.4 µm, *a*_y_ = 0.5 µm, and *a*_z_ = 2 µm. The spectra are cut at the wavelength of diffraction, which is varied with the angle of incidence and λ_d_ ≈ 3.5, 4.1, and 4.7 µm at angles of 0°, 10°, and 20°, respectively. (**b**) Maps of the *E*_x_ and *E*_z_ field components in resonant states. The XZ coordinate plane is shown for nanoantenna cross-sections.

Furthermore, nanoantennas with *a*_x_ = 2.4 µm are analyzed with oblique incidence with TM polarization to demonstrate the emergence of a resonant response related to *n*_x_ ≥ 2 (Figure 3a,b). The resonant mode at λ = 3.7 µm with θ = 0° (normal incidence of the plane wave) is significantly distinct from the ones corresponding to the resonant modes at λ = 4.5 µm and θ = 10° of oblique incidence. A similar effect is observed for λ = 4.9 µm and θ = 20°. These two resonant modes are associated with *n*_x_ = 2 and are associated with a decrease in reflectance.

## 4. Lattice and Dispersion Effects

Resonances in the nanoantenna array are defined not only by the cuboid dimensions and excitation conditions but also by the arrangement of the nanoantennas in the lattice and material dispersion. In this section, the excitations of multipolar nanoantenna resonances are shown to be determined by the lattice period, and a change in the array period is demonstrated to effectively shift the resonances from the ones excited in the closely spaced array. Nanoantenna resonances in the case of hBN dispersion are also analyzed with realistic material losses resulting from the phonon-polariton excitations.

### 4.1. Collective Resonances

The optical response of the rectangular lattice arrangement has been extensively researched in both plasmonic and high-refractive-index nanoantennas [94,95,96,97,98,99,100]. The possibility of separating resonances by increasing the lattice period and exciting lattice resonances is a general property of nanoantennas with multipolar resonances in periodic arrays. The degree to which a resonance with reasonable width can be excited in the spectral domain is mainly defined by the strength of the single-nanoantenna resonance (how large the effective polarizability of an electric/magnetic dipole, quadrupole, etc. is) and losses in the structure. Thus, one can expect that the naturally occurring hyperbolic materials with low losses and strong resonance in nanoantennas, such as hBN patterns, can support lattice resonances in a broad spectral range.

**Figure 4:**
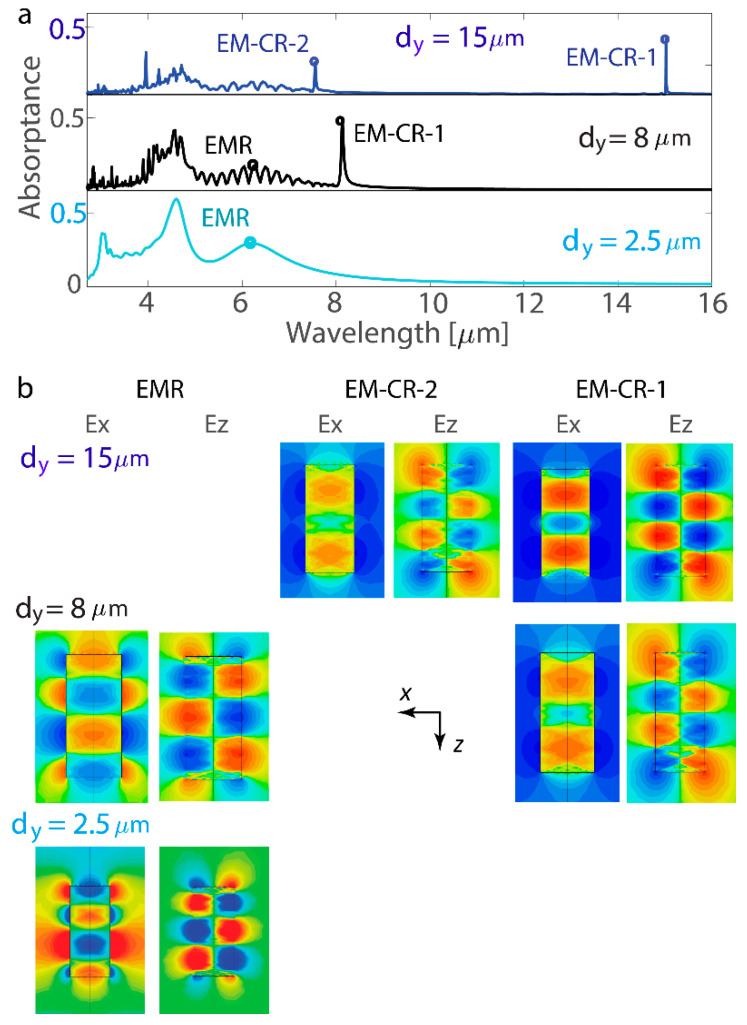
Collective resonances in the nanoantenna array. (**a**) Absorptance spectra for the metasurface with the collective response (CR). In the metasurface with d_y_ = 8 µm, the electric multipolar collective response (EM-CR) is at λ_1_ ≈ d_y_ = 8 µm. In the metasurface with d_y_ = 15 µm, two EM-CRs are observed at λ_1_ = d_y_= 15 µm and λ_2_ = d_y_/2 = 7.5 µm. The metasurface has an arrangement of *d*_x_ = 2.5 µm, and the nanoantennas have the dimensions *a*_x_ = 0.9 µm, *a*_y_ = 0.5 µm, and *a*_z_ = 2.0 µm. (**b**) Maps of the electric components *E*_x_ and *E*_z_ when the nanoantenna is in a resonant state. The XZ coordinate plane is shown for the nanoantenna cross-sections. EMRs are observed for *n*_z_ = 3, and EM-CRs are excited for *n*_z_ = 4.

A strong collective resonant mode is observed emerging when the single-nanoantenna resonance is spectrally located near the wavelength of diffraction and effectively couples to the Rayleigh anomaly. Similarly, a nanoantenna made of a hyperbolic medium can possess resonances at wavelengths determined by the pitch of the nanoantenna array. Figure 4a demonstrates the array response in two cases: when the metasurface with *d*_y_ = 8 µm has an electric multipolar collective response (EM-CR) at wavelength λ_1_ ≈ *d*_y_ = 8 µm and when the metasurfaces with *d*_y_ = 15 µm have two EM-CRs at λ_1_ = *d*_y_ = 15 µm and λ_2_ = *d*_y_/2 = 7.5 µm. Analyzing the map of the resonant mode, one can observe that EM-CRs have a larger *n*_z_ than EMR (Figure 4b). In additional simulations not presented here, the resonant modes at λ_3_ = *d*_y_/3 and λ_4_ = *d*_y_/4 are excited. An impressive characteristic of the collective resonant response is its ability to be excited at significant spectral separation from the resonant response of the individual nanoantenna. The spectral profile in Figure 4a demonstrates distinct collective modes, spectrally separated by 8 µm from the reddest EMR in the tightly packed nanoantenna lattice. Thus, when the nanoantenna array is the sparser lattice with periodicity *d*_y_, high-quality-factor collective resonant responses emerge at wavelengths *d*_y_, *d*_y_/2, *d*_y_/3, and so on.

Collective resonances introduce a new dimension of control, allowing metasurfaces to sustain resonances within a spectral range dictated by the array’s periodicity. Both the shape of the nanoantenna and its arrangement within the lattice significantly influence resonance excitation, leading to the lattice Kerker effect. For this effect to manifest, the periodicity of the array must be on the order of the resonance wavelength of the individual nanoantenna (or the resonance wavelength of the tightly packed array). This results in lattice resonances being excited when the wavelength is comparable to the periodicity, or in proximity to the Rayleigh anomaly. The geometric parameters of the cuboids are crucial for tuning this effect, as their dimensions must be optimized to align the nanoantenna resonances with the Rayleigh anomaly. In the case illustrated in Figure 4, where the effective resonance wavelength of the tightly packed nanoantenna lattice is slightly greater than 6 µm, the strongest lattice resonance is anticipated for a period ranging from 6 to 9 µm, being excited at a wavelength very close to the array period.

### 4.2. hBN Dispersion

**Figure 5:**
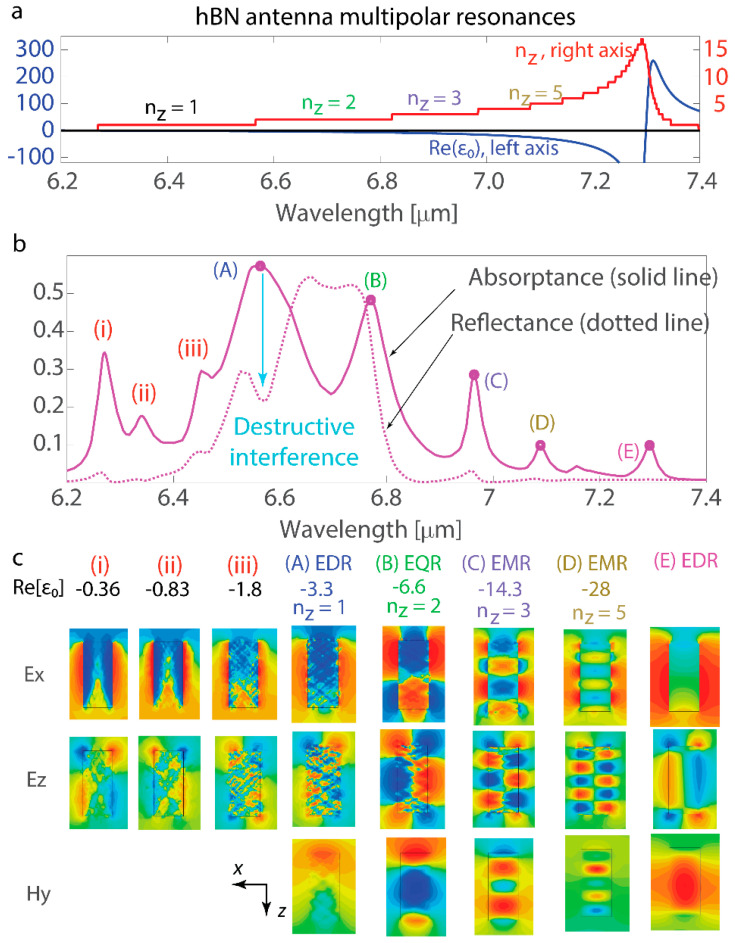
Multipolar resonances in the hBN nanoantenna. (**a**) In-plane tensor term of hBN ε_0_ = ε_x_ = ε_y_ and *n*_z_ calculated with Equation (2) for the case of *n*_y_ = 0 and *n*_x_ = 1. (**b**) Absorptance and reflectance spectra (solid and dotted lines, respectively) of the periodic array of the hBN nanoantenna. At the spectral position of (A) EDR, one can see a pronounced decrease in reflectance resulting from the destructive interference of multipolar resonances. The metasurface has an arrangement of *d*_x_ = *d*_y_ = 2.5 µm, and the nanoantennas have dimensions of *a*_x_ = 0.9 µm, *a*_y_ = 0.5 µm, and *a*_z_ = 2 µm. (**c**) *E*_x_, *E*_y_, and *H*_y_ field distributions in multipolar resonant states. The XZ coordinate plane is shown for nanoantenna cross-sections.

Multipolar resonances in nanoantennas were considered taking into account the hBN dispersion and the large variations of the constituent material permittivity. To account for realistic dispersion and material losses, the resonant modes and scattering characteristics were examined for cuboid nanoantennas with hBN permittivity within the band 6.2–7.4 µm and the data and model from [82]. The real part of the in-plane tensor term of the hBN permittivity shifts from null to significantly negative, with a subsequent sharp rise in the positive values (Figure 5a). The calculations confirm that the model presented by Equation (2) adequately predicts the types of excited resonances, and one can see several EDRs along with the full range of EMRs with *n*_z_ ranging from 2 to 5 (Figure 5b,c).

In the periodic array, the reflectance is relatively low at 6.2 µm < λ < 6.45 µm and 6.8 µm < λ < 7.4 µm. However, the EDR (A) and EQR (B) resonances are the strongest (see absorptance maxima in Figure 5b), and their out-of-phase interference in the wavelength range between resonances causes the highest reflectance (6.6 µm < λ < 6.8 µm). The overlap of multiple resonances around λ ≈ 6.55 µm causes a significant drop in reflectance; even though strong EDR resonances are excited and absorptance is significantly increased, the destructive interference of resonances results in the near-zero reflectance of the nanoantenna array (compare the profiles of reflectance and absorptance in Figure 5b).

**Figure 6:**
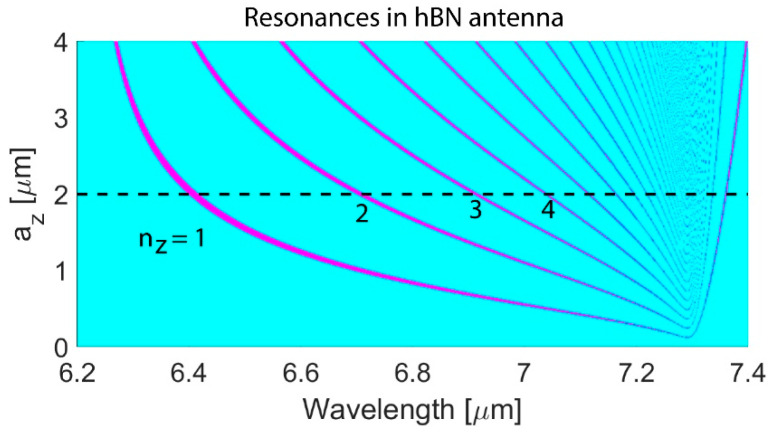
*n*_z_ obtained from Equation (2) of the variable *a*_z_ for the hBN nanoantenna. The dimensions of the nanoantenna are *a*_y_ = 0.5 µm and *a*_x_ = 0.9 µm, and the excitation is with *n*_y_ = 0 and *n*_x_ = 1. The dashed black line corresponds to *a*_z_ = 2 µm.

Using Equation (2), *n*_z_ is calculated for a wide range of nanoantenna *a*_z_ in the entire spectral range of the hBN dispersion (Figure 6). For larger nanoantennas, low-order resonances are excited at the shorter wavelength; for example, nanoantennas with *a*_z_ = 4 µm are expected to have EQR and MR (denoted ‘2’ in Figure 6 because the resonances correspond to *n*_z_ = 2) resonances at λ ≈ 6.45 µm, while nanoantennas with *a*_z_ = 1 µm support the excitation of these resonances at λ ≈ 7.1 µm.

### 4.3. hBN Nanoantenna Pair

**Figure 7:**
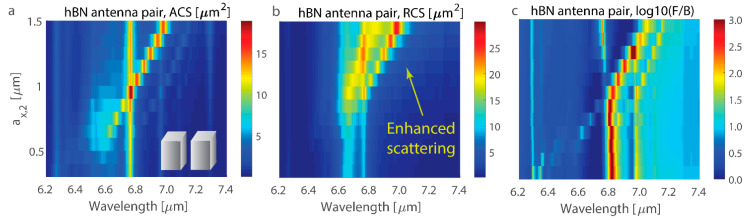
Absorption and scattering cross-sections of the hBN nanoantenna pair: (**a**) Absorption cross-section (ACS), (**b**) scattering cross-section (RCS), and (**c**) the ratio of forward to backward scattering of the nanoantenna pair (F/B, on a logarithmic scale). Both nanoantennas have dimensions of *a*_y,1_ = *a*_y,2_ = 0.5 µm and *a*_z,1_ = *a*_z,2_ = 2 µm, but one has *a*_x,1_ = 0.9 µm, and another has *a*_x,2_, which is varied. The *y*-axis is the same for all three plots. The nanoantenna centers have the same coordinates in the *y*- and *z*-axes, and the side-to-side gap on the *x*-axis is 0.5 µm.

Control of the array absorptance and scattering properties is demonstrated for the hBN nanoantenna pair. Two hBN nanoantennas are considered with all except one dimension of both nanoantennas, being fixed to *a*_y,1_ = *a*_y,2_ = 0.5 µm, *a*_z,1_ = *a*_z,2_ = 2 µm, and *a*_x,1_ = 0.9 µm, and *a*_x,2_ of the second nanoantenna is varied (Figure 7). The cross-section of total absorption indicates the positions of the resonance in the hBN nanoantenna pair (Figure 7a), and one can see that increasing *a*_x,2_ of the second nanoantenna effectively shifts all its resonances to the red side of the spectrum. At the spectral point where resonances overlap, the total scattering cross-section significantly increases (Figure 7b) because of the greater number of possible scattering channels and their interference (often referred to as superscattering [101]). Similarly, the forward-to-backward scattering ratio drastically increases on the red side of resonance overlap and reaches the value of 10^3^–10^4^ (Figure 7c; the logarithmic scale is limited to 3 for clarity). Among others, a high forward-to-backward scattering ratio is observed at resonant values of the total scattering cross-section, indicating that resonant in-phase interference suppresses scattering in the backward direction in analogy to the Kerker effect in plasmonic and all-dielectric structures.

Exploring the van der Waals nanoantenna with hyperbolic dispersion within the band 6.2–7.4 µm, one can observe that a large number of resonances are possible, including magnetic resonance. Resonances are defined by the geometrical parameters and permittivity of the nanoantenna according to Equation (2), and the light scattering from the nanoantenna depends on resonance overlap and interplay. In particular, one can achieve superscattering and resonant directional scattering with a forward-to-backward ratio as high as several thousand.

## 5. Conclusions

To sum up, nanoantennas made of a hyperbolic medium were demonstrated to support strong multipolar resonant excitations resulting from the propagating high-k modes of the hyperbolic medium and their reflection from the nanoantenna edges. For the cuboid nanoantenna, multipolar modes were shown to be accurately predicted by the analytical equations depending on the cuboid dimensions and material permittivity. The analytical model effectively predicts the modes of cuboid nanoantennas, which form high-quality-factor cavities with minimal radiative loss due to strong high-k wave reflections. Among the resonances supported by the nanoantenna, there are electric quadrupolar modes, which are associated with magnetic resonant excitations in the nanoantenna, analogous to what happens in high-refractive-index nanoantennas. Resonant multipolar modes of the nanoantenna result in a decrease in the reflectance of the nanoantenna lattice to negligible values, which is the generalized resonant Kerker effect. The collective resonances offer an extra level of control and the potential for metasurfaces to support resonances in the spectral range determined by the array periodicity.

The analysis of resonant metasurfaces with van der Waals hyperbolic nanoantennas reveals how multipolar excitations can be finely tuned through dimensional adjustments, leading to precise control over light behavior. The interplay of these excitations enables the manipulation of the light confinement to unprecedented levels, enhancing the overall optical response of the metasurfaces. This work emphasizes the significance of leveraging hyperbolic materials to achieve extreme light confinement and directional control, advancing our understanding of nanoscale light–matter interactions. By pushing the boundaries of resonance engineering, this research contributes to the broader goal of mastering light manipulation at the fundamental level.

## Data Availability

The data presented in the author’s plots are available upon request from the author. The data are not publicly available due to technical, resource, and time constraints.
